# Chemical Profile and Bioactivity Evaluation of *Salvia* Species from Eastern Europe

**DOI:** 10.3390/antiox12081514

**Published:** 2023-07-28

**Authors:** Simon Vlad Luca, Krystyna Skalicka-Woźniak, Cosmin-Teodor Mihai, Adina Catinca Gradinaru, Alexandru Mandici, Nina Ciocarlan, Anca Miron, Ana Clara Aprotosoaie

**Affiliations:** 1 Biothermodynamics, TUM School of Life Sciences, Technical University of Munich, 85354 Freising, Germany; 2 Faculty of Pharmacy, “Grigore T. Popa” University of Medicine and Pharmacy of Iasi, 700115 Iasi, Romania; 3 Department of Natural Products Chemistry, Medical University of Lublin, 20-093 Lublin, Poland; 4 Advanced Research and Development Center for Experimental Medicine (CEMEX), “Grigore T. Popa” University of Medicine and Pharmacy of Iasi, 700454 Iasi, Romania; 5 Botanical Garden, Academy of Sciences of Moldova, 2002 Chisinau, Moldova

**Keywords:** sage, Lamiaceae, antioxidant, antimicrobial, cytotoxicity, LC-HRMS/MS, multivariate analysis

## Abstract

The *Salvia* genus comprises about 1000 species endowed with medicinal, aromatic, cosmetic, and ornamental applications. Even though the genus is one of the most-studied taxa of the Lamiaceae family, data on the chemical composition and biological properties of certain locally used *Salvia* species are still scarce. The present work aimed to evaluate the phytochemical profile and antimicrobial, antioxidant, and cytotoxic potential of ten *Salvia* species that grow in Eastern Europe (e.g., the Republic of Moldova). LC-HRMS/MS metabolite profiling allowed for the annotation of 15 phenolic and organic acids, 18 flavonoids, 19 diterpenes, 5 sesterpenes, and 2 triterpenes. Multivariate analysis (e.g., principal component analysis, hierarchical cluster analysis) revealed that *S. austriaca*, *S. nutans*, and *S. officinalis* formed individual clusters, whereas the remaining species had a similar composition. *S. officinalis* showed the highest activity against *Staphylococcus aureus* and *Streptococcus pneumoniae* (MIC = 0.625 mg/mL). As evaluated in DPPH, ABTS, and FRAP assays, *S. officinalis* was one of the most potent radical scavenging and metal-reducing agents (CE_50_ values of 25.33, 8.13, and 21.01 μg/mL, respectively), followed by *S. verticillata*, *S. sclarea*, *S. kopetdaghensis*, *S. aethiopis*, and *S. tesquicola.* Pearson correlation analysis revealed strong correlations with rosmarinic acid, luteolin-*O*-glucuronide, and hydroxybenzoic acid. When the cytotoxic activity was evaluated in human breast carcinoma MCF-7 and MDA-MB-231 cells, no significant reduction in cell viability was observed over the concentrations ranging from 25 and 100 μg/mL. The results confirm the potential use of understudied *Salvia* species as promising sources of antioxidant compounds for developing novel pharmaceutical, nutraceutical, or cosmeceutical products.

## 1. Introduction

*Salvia* is one of the largest genera of the Lamiaceae family, consisting of about 1000 species. It includes medicinal, aromatic, culinary, and ornamental plants with many pharmaceutical, food, and cosmetic applications. Although *Salvia* plants are distributed worldwide, they are predominantly found in temperate and tropical areas (Mediterranean region, Central and South-East Asia, and Central and South America) [1,2]. For a long time, sage species have been traditionally used for their carminative, spasmolytic, antiseptic, astringent, wound-healing, and anti-inflammatory properties [3,4]. In European folk medicine, sage has been used to treat gastrointestinal disorders (dyspepsia, flatulence, abdominal spasms, diarrhea, inflammation of intestinal mucosa), inflammation of the mouth and throat, excessive sweating, coughs, skin inflammations, and galactorrhea [3,4]. In Asia and South America, sage plants have been used to treat various complaints such as rheumatism, gout, ulcers, diarrhea, and hyperglycemia [5]. Although *Salvia officinalis* (common sage, Dalmatian sage) is the most common representative of the genus, many other *Salvia* species (*S. fruticosa*, *S. lavandulifolia*, *S. sclarea*, *S. tomentosa*) are important for the production of essential oils, pharmaceuticals, colorants, cosmetics, perfumes, and biocides [4]. *S. miltiorrhiza* (Danshen, red sage) is a popular traditional Chinese medicinal product indicated for treating cardiovascular diseases. Furthermore, *S. hispanica* (Chia) is largely used in South America as an important nutraceutical [4].

Apart from their medicinal uses, some sage species, such as *S. officinalis* and *S. fruticosa* (Greek sage), are also popular as culinary plants due to their flavoring, seasoning, and food preservative properties [5,6]. Numerous *Salvia* species have been extensively shown to possess various biological activities, including antioxidant, anti-inflammatory, neuroprotective, anticancer, and metabolic effects. All these emphasize the promising potential of sage plants and their compounds in developing health-promoting agents. Apart from essential oil and non-volatile terpenes, *Salvia* species are valuable sources of polyphenols that significantly contribute to their bioactivity. However, the chemical composition is variable depending on genetic and environmental factors. In this regard, data on the chemical composition and associated bioactivity of some locally used *Salvia* species are still scarce. In the Republic of Moldova, the genus *Salvia* is represented by 12 species [7], with *S. officinalis*, *S. nemorosa*, *S. nutans*, *S. pratensis*, and *S. aethiopis* being the most used in folk medicine [8]. Although some Moldavian sage species (*S. officinalis, S. sclarea*) have been investigated concerning the chemistry of their essential oils [7,9], the non-volatile composition and biological properties have been poorly studied. To the best of our knowledge, only one study focused on the polyphenolic composition and antioxidant activity of six *Salvia* species from spontaneous Moldavian flora [10]. This work aimed to evaluate the phytochemical profile of polyphenolic compounds and non-volatile terpenes of ten Moldavian *Salvia* species from ex situ cultures. In addition, the antioxidant, antimicrobial, and cytotoxic activities were assessed. 

## 2. Materials and Methods

### 2.1. Chemicals

Gallic acid, 2,2-diphenyl-1-picrylhydrazyl radical (DPPH), 2,2′-azino-bis(3-ethylbenzothiazoline-6-sulfonic acid) diammonium salt (ABTS), potassium ferricyanide, iron (III) chloride, acetonitrile, and formic acid were purchased from Sigma-Aldrich (Steinheim, Germany). Folin–Ciocalteu’s phenol reagent, 3-(4,5-dimethyl-2-thiazolyl)-2-5-diphenyl-2H-tetrazolium bromide (MTT), dimethyl sulfoxide (DMSO), ethanol, and methanol were from Merck (Darmstadt, Germany). Trichloroacetic acid and potassium persulfate were supplied by Riedel-de-Haën (Seelze, Germany). Ultrapure water was obtained using an SGWater Ultra Clear TWF water purification system (Siemens Water Technologies Corp., Warrendale, PA, USA).

### 2.2. Plant Material and Extraction

**Figure 1:**
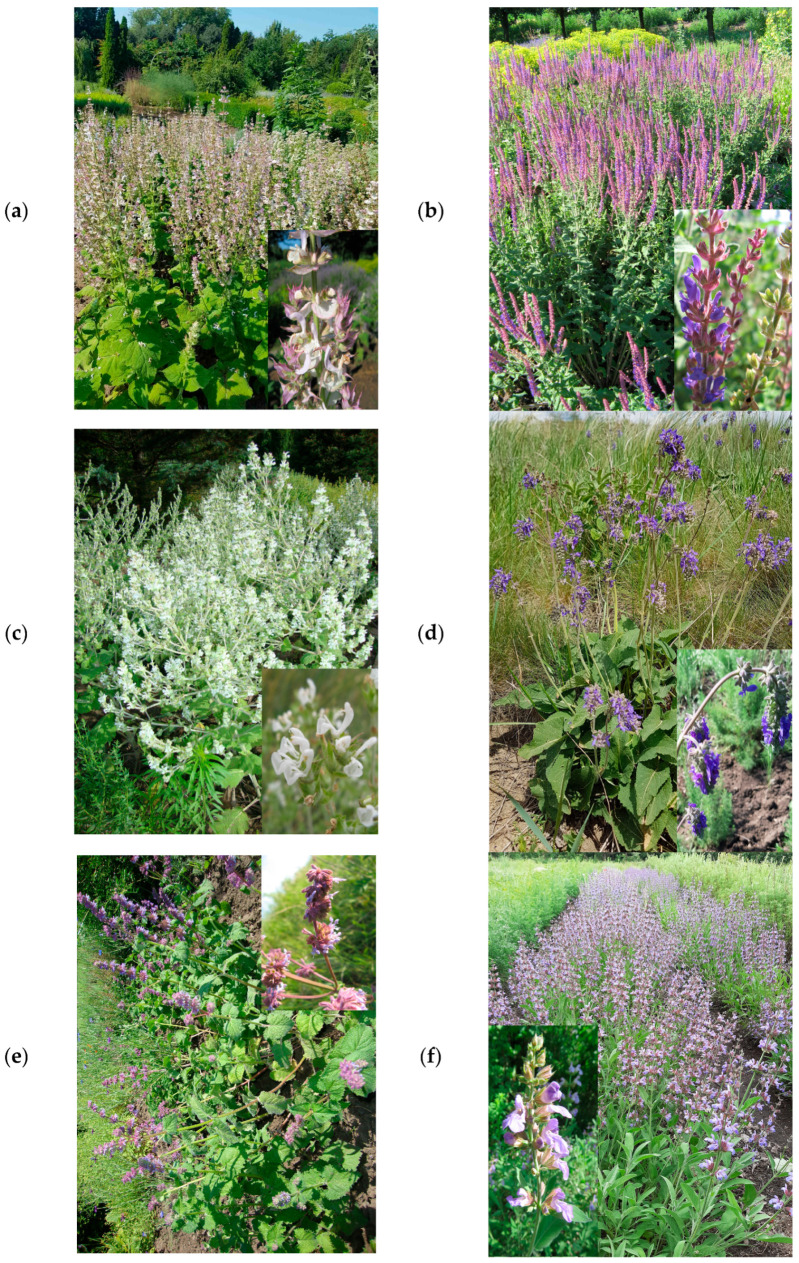

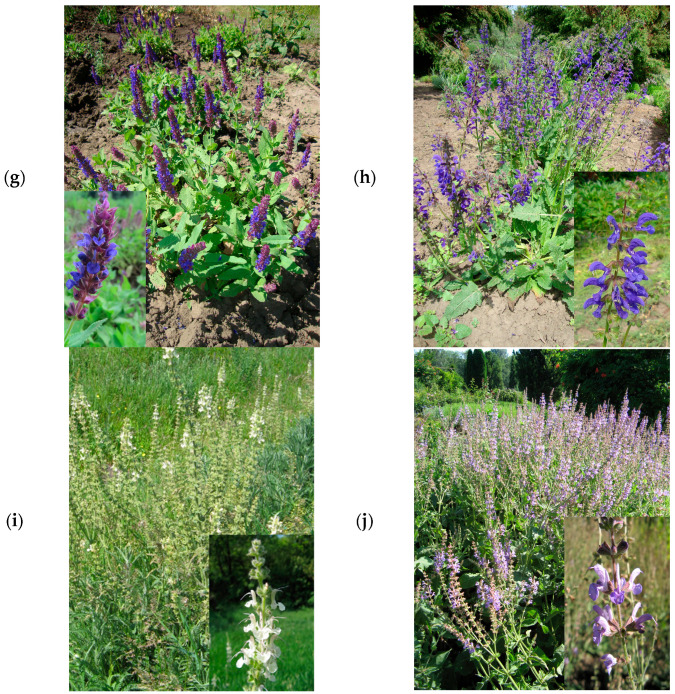
*Salvia* species: (**a**) *Salvia sclarea* L., (**b**) *Salvia tesquicola* Klok. and Pobed., (**c**) *Salvia aethiopis* L., (**d**) *Salvia nutans* L., (**e**) *Salvia verticillata* L., (**f**) *Salvia officinalis* L., (**g**) *Salvia nemorosa* L., (**h**) *Salvia pratensis* L., (**i**) *Salvia austriaca* Jacq., (**j**) *Salvia kopetdaghensis* Kudr.

The aerial parts of the ten *Salvia* species (Figure 1) were harvested from the crop fields of the National Botanical Garden ‘Alexandru Ciubotaru’ Chisinau, Republic of Moldova (GPS: N 46°58′25.43″, E 28°52′47.16″), during the flowering period (July 2019). Voucher specimens (Table 1) were deposited in the Department of Pharmacognosy and Phytotherapy, *Grigore T. Popa* University of Medicine and Pharmacy Iasi (Romania). The air-dried and powdered aerial parts (4 g) were extracted under reflux with 100 mL of 80% ethanol for 2 h at 60 °C. The extracts were concentrated in a rotary evaporator (Büchi Rotavapor, Flawil, Switzerland) under reduced pressure at 40 °C, and then they were stored in a freezer at −18 °C until analysis. 

### 2.3. Total Phenolics Quantification

The total phenolic content (TPC) of extracts from selected *Salvia* species was determined with Folin–Ciocalteu reagent according to the method of Singleton, with slight modification [11,12]. In this regard, suitable dilutions in 80% ethanol were prepared for each extract. Gallic acid was used as a reference standard, and TPC was expressed as mg gallic acid equivalents/g dry extract (mg GAE/g extract).

### 2.4. LC-HRMS/MS Analysis

The LC-HRMS/MS analysis was performed on an Agilent 1200 HPLC (Agilent Technologies, Palo Alto, CA, USA) connected to a quadrupole-time-of-flight MS detector (G6530B). The HPLC separation was accomplished on a Phenomenex Gemini C18 column (100 mm × 2 mm i.d., 3 μm) operated at 20 °C. A linear gradient elution (10–60% B in 0–45 min) was achieved using 0.1% formic acid in water (A) and 0.1% formic acid in acetonitrile (B) at a flow rate of 0.2 mL/min. The sample injection volume was 10 μL. The detection was carried out in negative electrospray ionization mode, with the spectra recorded in the range of *m*/*z* 100–1000 Da. The ion source parameters were as follows: carrier gas flow rate—10 L/min; carrier gas temperature—275 °C; sheath gas flow rate—12 L/min; sheath gas temperature—325 °C; nebulizer pressure—35 psi; capillary, fragmentor, skimmer, and octapole radiofrequency voltages—4000 V, 140 V, 65 V, and 750 V, respectively. MS/MS fragmentation was carried out via automated fragmentation, with the collision-induced dissociation energy set at 30 V. Mass Hunter software version B.08.00 (Agilent Technology) was used for data acquisition and processing, including the prediction of chemical formula and exact mass calculation. 

### 2.5. Antimicrobial Assay

#### 2.5.1. Microbial Strains

The antimicrobial activity of *Salvia* extracts was tested against standard strains (American Type Culture Collection-ATCC) of Gram-positive bacteria (*Staphylococcus aureus* ATCC 25923, *Streptococcus pneumoniae* ATCC 49619), Gram-negative bacteria (*Escherichia coli* ATCC 25922, *Pseudomonas aeruginosa* ATCC 27853), and one pathogenic fungus (*Candida albicans* ATCC 10231). They were provided by Liofilchem (Abruzzi, Italy).

#### 2.5.2. Minimum Inhibitory Concentration (MIC)

The broth microdilution method was used to determine MIC values according to the CLSI (Clinical and Laboratory Standards Institute) guidelines [13]. Serial double dilutions of the *Salvia* extracts ranging from 10 to 0.03 mg/mL were prepared in Mueller–Hinton broth (Biolab Zrt., Budapest, Hungary) using 24-well cell cultures plates (Becton Dickinson Labware Europe, Le Pont De Claix, France). For *Streptococcus pneumoniae*, the Mueller–Hinton broth was supplemented with 5% (*v*/*v*) lysed horse blood (Oxoid, Basingstoke, UK), and for *Candida albicans*, Sabouraud dextrose agar medium (Liofilchem, Abruzzi, Italy) was used; 10 μL of inoculum (10^5^ CFU/well) were added to each well. The plates were incubated for 24 h, at 35 °C. The growth of microorganisms was monitored by visual assessment of turbidity. Negative controls (microbial strains growth control, sterility control, solvents used to dilute the extracts) were also included in the assay. Stock solutions (20 mg/mL) of extracts in DMSO–ultrapure water (5:5, *v*/*v*) were prepared for antimicrobial testing. The MIC is defined as the lowest concentration of extract that inhibits the growth of the tested microbial strains [14].

### 2.6. Antioxidant Assays

Solutions stock of *Salvia* extracts (20 mg/mL) in 80% ethanol were prepared and adjusted at suitable working concentrations in each test. All antioxidant assays were performed in triplicate.

#### 2.6.1. DPPH Radical-Scavenging Assay

The test was carried out using the method described by Malterud et al. [15]. First, 2.95 mL of DPPH solution in methanol (A_517 nm_ = 1.02 ± 0.03) was mixed with 0.5 mL of *Salvia* extract dilutions at different concentrations (20.83–333.33 μg/mL). The absorbance was measured at 517 nm before adding the extract dilution (A_0_) and after 5 min reaction time (A_end_). The percentage of DPPH scavenging activity of each extract was calculated as follows: *DPPH scavenging activity (%) = 100 × [(A_0_ – A_end_)/A_end_]*. Gallic acid (1.30–333.33 μg/mL) was used as the positive control.

#### 2.6.2. ABTS Radical-Cation-Scavenging Assay

The test was performed according to the method of Re et al. [16]. The ABTS radical cation was obtained by incubating ABTS stock solution (7 mM) with potassium persulfate (2.45 mM) at room temperature in the dark for 16 h before use. Then, the ABTS radical-cation solution was diluted with ethanol to yield an absorbance of 0.70 ± 0.02 at 734 nm. Then 0.02 mL of each dilution of *Salvia* extract (12.5–100 μg/mL) was mixed with 1.98 mL ABTS radical cation solution. The absorbance at 734 nm was determined after a 6 min reaction time. The capacity to scavenge ABTS radical cation was determined using the following equation: *ABTS scavenging activity (%) = 100 × [(A_control_ – A_sample_)/A_sample_]*. Gallic acid (0.39–100 μg/mL) was used as the positive control.

#### 2.6.3. Ferric Ion Reducing Antioxidant Power Assay (FRAP)

The capacity of *Salvia* extracts to reduce iron (III) to iron (II) was evaluated using the Oyaizu method [17] with minor changes. First, 0.5 mL of *Salvia* extract dilutions (16.69–267.55 μg/mL) was mixed with 1.2 mL of 0.2 M phosphate buffer (pH 6.6) and 1.25 mL of 1% potassium ferricyanide and incubated at 50 °C for 20 min. Then, 1.25 mL of 10% trichloroacetic acid was added to it. The mixture was centrifuged at 3000 rpm for 10 min, and after that, 1.25 mL of the upper layer was treated with 1.25 mL of ultrapure water and 0.25 mL of 0.1% ferric chloride. The absorbance was measured at 700 nm after 90 s. Gallic acid (0.24–3.84 μg/mL) was used as the positive control. A high absorbance value indicated the potent reducing capacity of the samples.

### 2.7. Cell Viability Assay

#### 2.7.1. Cell Lines

Human breast carcinoma MCF-7 (ATCC, HTB-22) and MDA-MB-231 (ATCC, CRM-HTB-26) cell lines were maintained in DMEM (Dulbecco’s modified Eagle medium, Biochrom AG, Berlin, Germany), supplemented with 10% FSB (fetal bovine serum, Sigma, Steinheim, Germany), 100 IU/mL penicillin (Biochrom AG, Berlin, Germany), and 100 µg/mL streptomycin (Biochrom AG, Berlin, Germany) at 37 °C in a humidified atmosphere of 5% CO_2_ in the air. The cell lines were a cordial donation of Prof. Charalambos Anastassiou from the University of Cyprus.

#### 2.7.2. MTT Assay

The cell viability was evaluated using the MTT assay [18]. Briefly, cells were seeded in 96-well plates (TPP Techno Plastic Products AG, Trasadingen, Switzerland) at a density of 5 × 10^3^ cells/well and allowed to attach and grow overnight. *Salvia* extracts were added to cell cultures in concentrations of 25–100 µg/mL, using as vehicle agent DMSO with a final concentration of 0.1%. After 48 h, the cells were washed and covered with 100 μL of fresh 10% FBS in DMEM. Then, 10 μL of MTT (5 mg/mL) was added to the medium, and cells were incubated for 3 h. DMSO was used to solve the formed formazan, and the absorbance was recorded at 570 nm (PG Instruments T70, PG Instruments Ltd., Lutterworth, UK). The assay was performed in five replicates. The cell viability (%) was calculated according to the formula: *% cell viability = [Absorbance_Sample_/[Absorbance_Control_] × 100*.

### 2.8. Data Analysis

Data are presented as mean ± standard deviation of the respective number of replicates. One-way analysis of variance with Tukey’s post hoc test was conducted; *p* < 0.05 was considered statistically significant. After Pareto scaling, the phytochemical data (peak areas from the base chromatograms) were imported into SPSS 20.0 software (IBM, New York, NY, USA) and used to perform principal component analysis, hierarchical cluster analysis, and Pearson correlation analysis. For antioxidant tests, the EC_50_ values were calculated by linear interpolation between values above and below 50% activity. In the FRAP assay, the EC_50_ value represents the concentration of extract/positive control that leads to an absorbance of 0.5.

## 3. Results and Discussion

### 3.1. Total Phenolic Content

The values of TPC are shown in Table 1. The highest amounts of polyphenols were determined in *S. officinalis* (126.91 mg GAE/g extract), followed by *S. sclarea* (110.90 mg GAE/g extract), *S. kopetdaghensis* (107.63 mg GAE/g extract), and *S. verticillata* (107.62 mg GAE/g extract). *S. nutans* and *S. austriaca* had the lowest contents of phenolic compounds among the investigated *Salvia* species (66.12 and 57.87 mg GAE/g extract, respectively). Our results align with the findings of other studies on *Salvia* phenolics. *S. verticillata*, *S. nemorosa*, and *S. aethiopis* from Turkey contained 167.1, 63.9, and 82.1 mg GAE/g extract, respectively [19]. Furthermore, *S. verticillata* from Serbia and *S. sclarea* from Iran contained outstanding amounts of total polyphenols (175.6 and 268 mg GAE/g extract, respectively) [20,21]. A previous study by Mocan et al. [22] showed TPC values of 65.02 mg GAE/ extract for *S. officinalis* from Romania. Hanganu et al. [10] reported lower values of TPC (22.25–118.75 mg GAE/g dry plant material) for six *Salvia* species from spontaneous Moldavian flora (*S. aethiopis*, *S. austriaca*, *S. nemorosa*, *S. nutans*, *S. sclarea*, *S. verticillata*). The provenience of plant material, the geographical and pedo-climatic factors that influence the growing and harvesting time, and the mode of results expression (extract/plant, reference standard) could explain the different outcomes. In our study, the plants were collected from field crops, while in the mentioned research, the plants were harvested from spontaneous flora. Mocan et al. [22] reported a similar trend for *S. transsylvanica* from Romania and pointed out that cultivated plants could produce higher levels of polyphenols than wild plants. In addition, abiotic factors such as rainfall, temperature, and cloud cover play significant roles in sage phenolics biosynthesis [23]. 

### 3.2. Metabolite Profiling Using LC-HRMS/MS

LC-MS platforms are extensively used to perform metabolite profiling, not only of common sage species but also of less-investigated or endemic *Salvia* species. Previously, the comprehensive phytochemical characterization of *S. officinalis* revealed the presence of more than 40 compounds, such as phenolic acids, flavonoids, diterpenes, and triterpenes [24]. *S. miltiorrhiza* Bunge was profiled using LC-MS, evidencing two main structural groups, namely, phenolic acids (monomers, dimers, trimers, and tetramers of hydroxycinnamic acids) and diterpenes (tanshinones) [25,26]. Shojaeifard et al. [27] documented the occurrence of flavonoids (e.g., rutin, luteolin-7-*O*-glucoside, apigenin-7-*O*-glucoside, cirsimaritin, eupatorin), phenolic acids (e.g., rosmarinic acid, salvianolic acid B), and diterpenes (e.g., carnosol) in 50 *Salvia* species collected from different regions of Iran, including *S. indica* L., *S. grossheimii* Sosn., *S nemorosa*, *S. palaestina* Benth., *S. spinosa* L., *S. syriaca* L., and *S. verticillata*. Zengin et al. [28] reported 66 compounds (phenolic acids, flavonoids, sugars, and fatty acids) in three *Salvia* species endemic to Turkey, namely, *S. blepharochlaena* Hedge and Hub.-Mor., *S. euphratica* var. *leiocalycina* (Rech.f.) Hedge, and *S. verticillata* subsp. *amasiaca* (Freyn and Bornm.) Bornm. *Salvia* species from Pakistan (*S. coccinea*, *S. lanata*, *S. moocroftiana*, *S. nubicola*, *S. plebeiana*) [29], Turkey (*S. veneris* Hedge, *S. poculata*, *S. eriophora* Boiss. and Kotschy, *S. ceratophylla* L., *S. sclarea*, *S. absconditiflora* Greuter and Burdet) [30,31,32,33], Greece (*S. pomifera* L., *S. fruticosa* Mill.) [34,35], and Poland (*S. przewalskii* Maxim., *S. cadmica* Boiss., *S. yangii* B.T. Drew, *S. abrotanoides* Kar.) [25,36,37] were also comprehensively characterized using LC-MS.

In the current study, the LC-HRMS/MS-based metabolite profiling of the ten *Salvia* species allowed for the annotation of 73 compounds belonging to 8 phytochemical classes. Total identification was performed by matching the spectro-chromatographic data with those obtained by standard injection, whereas partial identification was conducted by comparing the acquired data with those from databases (e.g., KNApSACK [38]) or relevant literature reporting on the LC-MS analysis of compounds from *Salvia* or Lamiaceae species [24,25,27,28,29,30,31,34,36,39,40,41,42,43]. The collected information (e.g., proposed identity, retention time, molecular formulas, fragment ions, sample distribution) is provided in Table 2. Overall, *S. aethiopis* showed the most complex profile (57 compounds), followed by *S. sclarea* (55 compounds) and *S. verticillata* (52 compounds). Next, 48 compounds were assigned in *S. austriaca* and *S. kopetdaghensis*, 46 compounds in *S. nutans*, 44 compounds in *S. tesquicola* and *S. pratensis*, 41 compounds in *S. officinalis*, and 39 compounds in *S. nemorosa*. To our knowledge, the LC-HRMS/MS-based phytochemical profiling of *S. nutans* and *S. kopetdaghensis* was performed herein for the first time, whereas a few studies have profiled *S. sclarea* [33], *S. aethiopis* [44], *S. verticillata* [27,45], *S. nemorosa* [27], *S. pratensis* [20], and *S. austriaca* [46]. 

Thirteen phenolic acids were labeled in the ten sage species, classified as hydroxybenzoic acids (**5** and **6**), hydroxycinnamic acids (**4**, **8**, **9**, **14**, **18**, and **34**), and hydroxycinnamic acid oligomers (**23**, **24**, **26**, **27**, and **29**). Dihydroxybenzoic acid (**5**) was present only in *S. nemorosa*, whereas feruloylmalic acid (**18**) was identified only in *S. aethiopis*. Furthermore, salvianolic acid H was annotated only in *S. pratensis*, while caffeoylthreonic acid (**9**) was distributed specifically in *S nemorosa* and *S. pratensis*. Caffeic acid-*O*-hexoside (**14**), a phenolic glycoside, was characteristic of *S. nutans* and *S. officinalis.* Interestingly, caffeic acid (**8**) and rosmarinic acid (**23**) were ubiquitously found in all species, while hydroxybenzoic acid (**6**) was absent only in *S. austriaca*. In a previous study [35], 10 phenolic acids, such as hydroxybenzoic, dihydroxybenzoic, caffeic, ferulic, vanillic, chlorogenic, neochlorogenic, cyrptochlorogenic, and rosmarinic acids, were reported in *S. fruticosa*. Salvianolic acid B, salvianolic acid K, and chlorogenic, caffeic, ferulic, coumaric, and rosmarinic acids were documented in *S. officinalis* [47]. Zengin et al. [28] reported at least ten phenolic acids (e.g., danshensu, caffeic acid, caffeic acid-*O*-hexoside, protocatechuic acid, coutaric acid, coumaric acid) in *S. blepharochlaena, S. euphratica* var. *leiocalycina*, and *S. verticillata* subsp. *amasiaca*. 

A series of 18 flavonoids was next labeled as flavanol aglycons (**22**), flavones aglycons (**31**, **36**, **41**, **48**), flavone glycosides (**10**–**12**, **15**, **17**, **20**, **21**, **25**, **28**, **30**, **33**), and flavonol glycosides (**13**, **16**). Apigenin-*O*-pentoside-*O*-hexoside (**10**), chrysoeriol-*O*-acetylglucuronides I (**30**), and II (**33**) were observed only in *S. nutans*. Luteolin-*O*-hexoside-*O*-glucuronide (**11**) was characteristic of *S. tesquicola*, whereas quercetin-*O*-hexoside (**13**) was found only in *S. officinalis*. Interestingly, luteolin-7-*O*-glucoside (**17**) and luteolin-*O*-glucuronide I (**20**) were retrieved in all ten sage species. Luteolin-*O*-acetylglucuronide (**28**) was found only in *S. nutans* and *S. verticillata*, while apigenin was distributed specifically in *S. sclarea* and *S. austriaca*. Luteolin-*O*-hexoside-*O*-rhamnoside (**15**) was present in *S. tesquicola*, *S. nutans*, and *S. officinalis*, whereas apigenin-7-*O*-glucoside (**21**) was specific to *S. sclarea*, *S. nemora*, and *S. austriaca*. Genkwanin (**48**), a methoxylated flavone, was distributed in *S. sclarea*, *S. pratensis*, and *S. austriaca*. Previously, flavonoids with similar structures were found in sage species [24,25,27,28,29,30,31,34,36,39,40,41,42,43]. 

With 19 congeners, diterpenes were the largest phytochemical class in *Salvia* spp. Most compounds were derivatives of rosmanol (**40**, **42**, **45**, **54**, **56**, and **65**) or carnosol (**37**, **47**, **52**, **54**, **55**, **59**, and **68**). *S. officinalis* was the only species that contained rosmanol II (**42**), methoxycarnosol (**54**), acetylhorminone II (**64**), and rosmaridiphenol (**65**). Salvipiliferol (**58**), hydroxysalviol (**62**), and salviol (**70**) were characteristic of *S. nemorosa*. Acetylhorminone I (**51**) was shown only in *S. verticillata*, whereas dihydroxycarnosic acid (**55**) was specifically found in *S. tesquicola* and *S. kopetdaghensis*. *S. sclarea* and *S. tesquicola* were the only species containing hydroxcarnosic acid I (**37**), whereas carnosol (**59**) was present only in *S. officinalis* and *S. kopetdaghensis*. Diterpenes are widely reported in the *Salvia* genus. For instance, Koutsoulas et al. [34] retrieved seven diterpenes in *S. fruticosa* (including rosmanol, carnosol, rosmadial, carnosic acid, methoxycarnosol) and two diterpenes in *S. pomifera*. Rosmanol, rosmaridiphenol, epirosmanol, epiisorosmanol, and methoxycarnosol were found in *S. veneris* [31], whereas carnosol, rosmanol, galdosol, carnosic acid, salviol, methyl carnosic acid, and 20-hydroxyfemiginol were shown in *S. officinalis* [24]. 

Five sesterpenes were putatively assigned as follows: lachnocalyxolides C (**43**), C’ (**46**), and A (**50**) in *S. nutans*; and salvimirzacolides I (**44**) and II (**57**) in *S. aethiopis.* Previously, lachnocalyxolides were identified in *S. lachnocalyx* Hedge [43], whereas salvimirzacolide was isolated from *S. mirzayanii* Rech. f. and Esfand. [38]. Nonetheless, oleanolic acid (**72**) and ursolic acid (**73**) were labeled as triterpenes in almost all *Salvia* species. Oleanolic acid (**72**) was absent in *S. sclarea*, *S. aethiopis*, *S. officinalis*, and *S. kopetdaghensis*, whereas ursolic acid (**73**) was not present in *S. sclarea*, *S. aethiopis*, *S. austriaca*, and *S. kopetdaghensis.* These triterpenes were previously reported in *S. pomifera*. and *S. fruticosa* [34]. Furthermore, two organic acids, namely, malic acid (**2**), quinic acid (**3**), and one sugar derivative, sucrose (**1**), were identified as non-specific metabolites in the polar region of the chromatograms (retention times <5 min, Table 2). In contrast, 13 fatty acid derivatives were found in the non-polar region of the chromatographic elution (retention times between 30 and 55 min, Table 2). 

**Figure 2:**
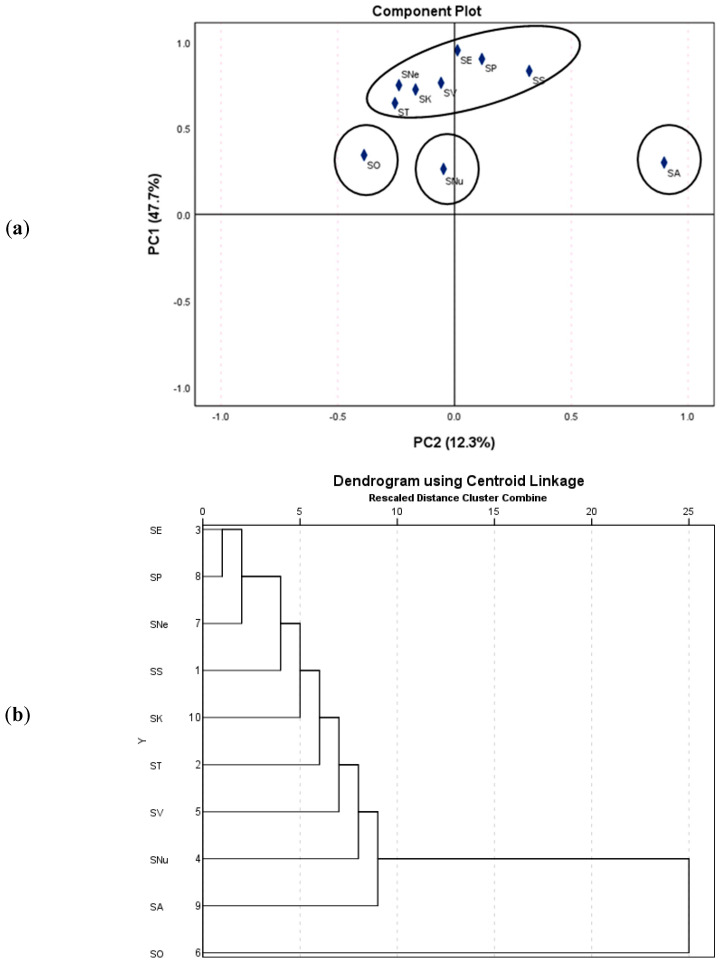
Overview of the phytochemical differences among the *Salvia* species based on LC-HRMS/MS data. Principal component analysis (**a**) and hierarchical cluster analysis (**b**) of *Salvia* species.

When it comes to the relative abundance of the constituents, it was found that rosmarinic acid (**23**) was the most predominant compound in *S. sclarea*, *S. tesquicola, S. aethiopis, S. verticillata, S. officinalis, S. nemorosa, S. pratensis,* and *S. kopetdaghensis*. Gallocatechin (**22**) was dominant in *S. nutans*, while salvianolic acid B (**35**) was abundant in *S. kopetdaghensis*. Luteolin-7-*O*-glucoside (**15**) was the major constituent in *S. aethiopis*, *S. nemorosa*, and *S. austriaca*. Caffeic acid (**8**) was found in relatively high levels in *S. aethiopis* and *S. nutans*. Luteolin (**31**), cirsimaritin (**41**), and apigenin (**36**) were significant in *S. sclarea* and *S. austriaca*. Carnosol (**59**), methylcarnosic acid (**68**), and rosmanol (**45**) were predominant in *S. officinalis*, whereas carnosic acid (**52**) and hydroxycarnosic acid (**47**) were found in high amounts in *S. tesquicola.* In an attempt to point out more objectively the chemotaxonomic differences between the ten sage species, a multivariate analytical approach based on principal component analysis and hierarchical cluster analysis was subsequently applied. The semi-quantitative data (peak areas of identified compounds after the Paretto scaling) were used as input information. As shown in the component plot (Figure 2a) and dendrogram (Figure 2b), *S. officinalis*, *S. austriaca*, and *S. nutans* formed individual clusters, whereas the remaining *Salvia* species could be regarding a single big cluster. 

### 3.3. Assessment of the Antimicrobial Activity

Screening plant sources to determine their antimicrobial properties is an important strategy to find new therapeutic anti-infective solutions and combat the multidrug-resistance phenomenon. It is estimated that about 75% of the pharmaceuticals used in anti-infective therapy are obtained from natural sources [48]. The extracts obtained from the ten sage species were evaluated concerning their antimicrobial properties against a standard panel of human pathogens, including Gram+ bacteria (*S. aureus* and *S. pneumoniae*), Gram− bacteria (*E. coli* and *P. aeruginosa*), and yeasts (*C. albicans*). The results (Table 3) showed that the MIC values of most samples were situated between 1.25 and 5 mg/mL. Only *S. officinalis* showed moderate activity (MIC = 0.625 mg/mL) when tested against *S. aureus* and *S. pneumoniae*. Furthermore, *Salvia sclarea* behaved more actively on the same bacteria (MIC = 1.25 mg/mL). Among the analyzed *Salvia* species, *S. nemorosa* and *S. pratensis* were the most active against *Candida albicans* (MIC = 1.25 mg/mL).

Previous literature reports revealed comparable antimicrobial properties of *Salvia* species. For instance, extracts from *S. pratensis* showed MIC values between 5 and 20 mg/mL against *S. aureus*, *P. aeruginosa*, *E. coli*, and *C. albicans* [20]. *S. verticillata* displayed MIC values between 1.25 and 20 mg/mL against a panel of eight bacterial and eight fungal strains [45]. *S. aethiopis, S. nemorosa*, and *S. sclarea* also showed MIC values between 1.25 and 20 mg/mL against *E. coli*, *K. pneumoniae*, *S. typhi*, *B. subtilis*, *S. epidermidis*, and *S. aureus* [49]. Mocan et al. [22] showed that extracts derived from *S. officinalis* exhibited MIC values between 0.01 and 0.18 mg/mL against *E. coli, P. aeruginosa, S. typhimurium, L. monocytogenes, E. cloacae, M. flavus, B. cereus,* and *S. aureus*. Gram-positive bacteria are more susceptible to *Salvia* extracts. The resistance of Gram-negative bacteria can be related to their multi-layered highly complex cell structure consisting of inner and outer membranes [48]. The outer membrane containing mainly lipopolysaccharides acts as an additional selective and impermeable barrier [50]. Although volatile terpenes are better known in terms of antimicrobial properties, many other specific metabolites of *Salvia* species (flavonoids, phenolic acids, diterpenes, triterpenes) are capable of inhibiting the growth of different pathogens affecting multiple targets of microbial cells. Flavonoids, such as apigenin, luteolin, and quercetin derivatives, may cause cell-membrane damage, and inhibition of nucleic-acid synthesis and of the bacterial respiratory chain [51]. Antimicrobial mechanisms of phenolic acids (rosmarinic, caffeic, ferulic acids) include damage to bacterial membrane integrity and bacterial cell morphology, leakage of cellular electrolytes, and alteration of microbial metabolism [52,53]. Sage diterpenes can inhibit microbial protein synthesis and damage microbial membrane structure and cellular respiration [54]. 

### 3.4. Assessment of Antioxidant Activity

Reactive oxygen species and oxidative stress are involved in many pathologies (cancer, cardiovascular and neurodegenerative diseases, skin disorders). The antioxidant abilities of plant products may be an important strategy to improve cell responses to injuries, counteract noxious and pathogenic stimuli, and preserve cell health status. The antioxidant capacity of the ten Moldavian *Salvia* species was assessed in three in vitro tests, namely, DPPH, ABTS, and FRAP. The results, presented as EC_50_ values (Table 4), show that the extracts obtained from *S. officinalis* and *S. verticillata* were the most potent DPPH radical scavengers (EC_50_ = 25.33 and 27.36 µg/mL, respectively). On the contrary, *S. nutans* and *S. austriaca* showed the weakest anti-radical activity. A similar trend was found in the ABTS assay when the EC_50_ values varied from 8.13 and 59.16 µg/mL. The following decreasing activity order can be concluded: *S. officinalis* > *S. verticilata* > *S. kopetdaghensis* > *S. pratensis* > *S. nemorasa* > *S. aethiopis* ~ *S. sclarea* > *S. tesquicola* >> *S. nutans* > *S. austriaca*. Lastly, the reducing power revealed that *S. kopetdaghensis* (EC_50_ = 19.75 µg/mL), *S. verticillata* (EC_50_ = 19.75 µg/mL), and *S. officinalis* (EC_50_ = 19.75 µg/mL) were the most active samples. 

Our results are comparable with those from the existing literature. For instance, CE_50_ values between 80.09 and 158.76 µg/mL in DPPH and 1.39 and 8.04 mol Trolox equivalents (TE)/mg in FRAP were reported for the extracts obtained from *S. aethiopis*, *S. austriaca*, *S. sclarea*, *S. nutans*, *S. verticillata*, and *S. nemorosa* [10]. Similarly, *S. officinalis*, *S. sclarea*, *S. pratensis*, *S. austriaca*, *S. nemorosa*, and *S. verticillata* displayed potent DPPH radical scavenging (53.44–189.94 μg TE/mL) and FRAP (1.19–5.89 μmol TE/100 mL) activities [55]. *S. verticillata* showed good antioxidant activity, as assessed in DPPH (EC_50_ = 33.04 μg/mL), ABTS (EC_50_ = 67.01 μg/mL), and NO (EC_50_ = 73.12 μg/mL) radical scavenging assays [45]. In addition, Matkowski et al. [56] reported potent DPPH radical scavenging (EC_50_ = 19.84 μg/mL), reducing power (0.671 g quercetin equivalents/g), and Trolox equivalent antioxidant capacity (13.30 mg TE/g) for *S. verticillata*. Similarly, the aerial parts extract of *S. pratensis* revealed EC_50_ values of 50.17 and 90.65 μg/mL in DPPH and ABTS radical scavenging tests, respectively [20]. Tohma et al. [44] also studied the antioxidant properties of *S. aethiopis* in DPPH, FRAP, and cupric-ion-reducing antioxidant (CUPRAC) assays. Extracts from *S. sclarea* were shown to scavenge DPPH (85.08 mg TE/g) and ABTS (33 mg TE/g) radicals, reduce ferric (77.06 mg TE/g) and cupric (144.75 mg TE/g) ions, and chelate ferrous ions (37.27 mg EDTAE/g) [57]. The antioxidant effects of other *Salvia* species (e.g., *S. blepharochlaena*, *S. euphratica* var. *leiocalycina*, *S. glutinosa*, *S. transsylvanica*, *S. syriaca*, *S. aegyptiaca*, *S. palaestina*, *S. absconditiflora*, *S. cadmica*, *S. ceratophylla*) are also documented in the literature [22,28,33,36,49,57,58]. 

**Figure 3:**
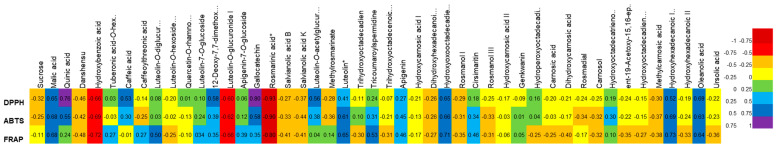
Correlation between specialized metabolites and antioxidant activity of *Salvia* species.

In an attempt to correlate the observed antioxidant activity (Table 4) with the phytochemical composition (Table 2), Pearson correlation analysis was subsequently performed. The peak areas from the LC-MS chromatograms were extracted and used as input information. As depicted in Figure 3, most compounds displayed negligible correlations (R values < 0.50). However, rosmarinic acid and, to a lesser extent, hydroxybenzoic acid and luteolin-*O*-glucuronide correlated well with the DPPH-radical-scavenging, ABTS-radical-scavenging, and metal-reducing activity. Rosmarinic acid and luteolin derivatives are particularly known to exert potent antioxidant effects, as revealed by numerous studies [59,60]. 

### 3.5. Assessment of the Cytotoxic Activity

Plant-derived products are highly valuable resources for developing chemopreventive and anticancer agents. Over 60% of anticancer drugs are obtained from natural products (plants, aquatic organisms, and microbial sources) [61]. *Salvia* species are a rich reservoir of many compounds with multiple bioactivities and have attracted great interest in screening cytotoxic agents. This section presents the influence of five selected sage species on the viability of human breast carcinoma MCF-7 and MDA-MB-231 cell lines. The MCF-7 cell line retains estrogen and progesterone receptors and is highly responsive to chemotherapy. MDA-MB-231 is a highly invasive and aggressive triple-negative breast cancer cell line [62,63]. The five *Salvia* species were chosen according to their use in Moldavian traditional medicine for different kinds of tumors [8]. Breast cancer, the most common malignancy in women [64], is also one of interest in the research of the cytotoxic potential of *Salvia* species and their metabolites. 

**Figure 4:**
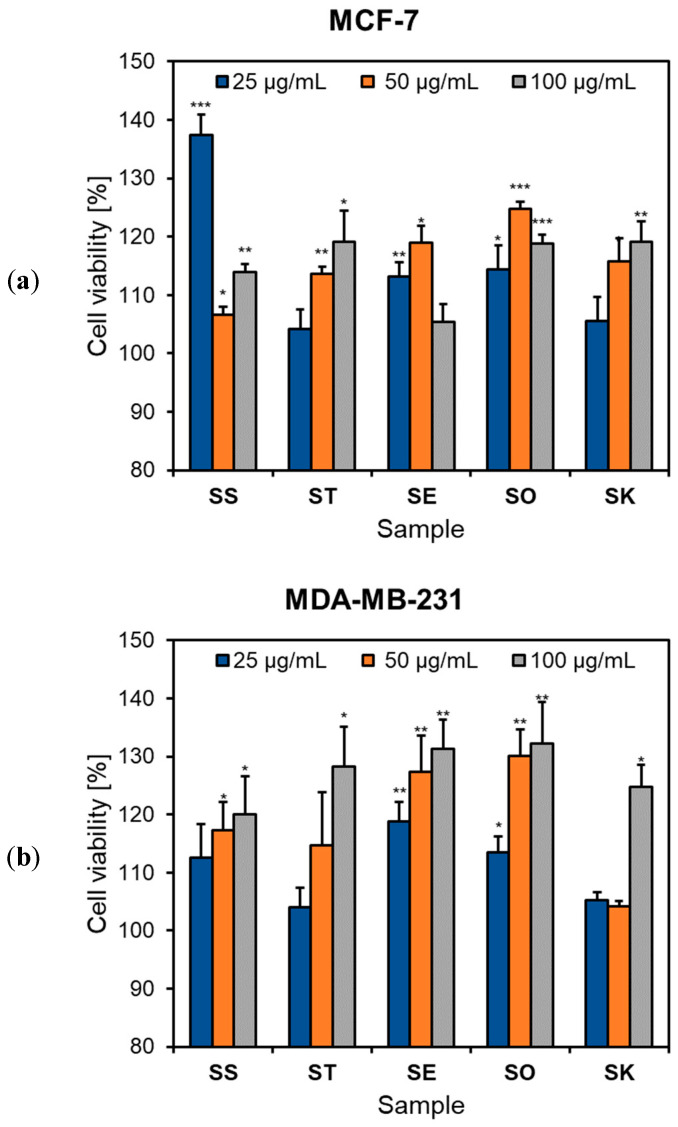
Influence of *Salvia* species on (**a**) MCF-7 and (**b**) MDA-MB-231 cell viability. Cells were incubated for 48 h with *Salvia* extracts (25–100 μg/mL), with the cell viability determined using MTT assay. Sample codes as in Table 1; * *p* < 0.05, ** *p* < 0.01, and *** *p* < 0.001 vs. negative control.

In our study, no sample showed a significant reduction in cell viability over the concentration domain ranging from 25 to 100 μg/mL (Figure 4). On the contrary, we found a slight increase in the number of viable cells at tested doses for all *Salvia* extracts. A similar effect was reported by Mocan et al. [22] in the case of Romanian *S. glutinosa* and *S. transsylvanica* at intermediary doses on MCF-7 and HepG2 cells. Furthermore, some previous studies revealed low cytotoxicity of the *Salvia* genus. For example, extracts derived from *S. verticillata* showed no viability reduction in epidermoid carcinoma A431, liver cancer HepG2, and colon carcinoma LoVo cells at concentrations of 5 to 50 μg/mL [45]. Similar outcomes were also reported for *S. pratensis*, with IC_50_ values for aerial part extracts >200 μg/mL in A431 cells [20]. Zengin et al. [28] confirmed the lack of antiproliferative activity of *S. verticillata* subsp. *amasiaca*, *S. euphratica* var. *leiocalycina*, and *S. blepharochlaena* in human alveolar lung epithelial carcinoma A549 and human breast adenocarcinoma MCF-7 cells. The cytotoxicity of eleven *Salvia* species (e.g., *S. aethiopis, S. nemorosa, S. syriaca*, etc.) was tested in MCF-7, acute promyelocytic leukemia HL60, and chronic myelogenous leukemia K562 cells, revealing IC_50_ values generally higher than 50 μg/mL [49]. Mocan et al. [22] showed a modest cytotoxic effect of *S. glutinosa* and *S. transsylvanica* against HepG2, A549, and MCF-7 cell lines with IC_50_ values greater than 100 μg/mL. Furthermore, Nicolescu et al. [58] evidenced IC_50_ values between 131.68 and 293.79 μg/mL for various extracts of *S. glutinosa* in MCF-7, HepG2, non-small cell lung carcinoma NCI H460, and cervical carcinoma HeLa cells. 

Although many studies have revealed good cytotoxic potential of certain sage species in lung (*S. hispanica*, *S. pilifera*, *S. macrosiphon*), prostate (*S. ballotiflora*, *S. hispanica*, *S. pilifera*), colon (*S. fruticosa*), and breast cancer (*S. officinalis*, *S. miltiorrhiza*, *S. fruticosa*, *S. verbenaca*, *S. atropatana*, *S. macrosiphon*, *S. rosmarinus*, *S. chloroleuca*) [5,65,66] there are, as we mentioned, opposite results. The type of extract (polar, lipophilic), the presence and concentration of specific components, the ratios between them, the tested concentration, and the exposure period significantly influence the outcome. Perhaps the identification and monitoring of some marker cytotoxic compounds would be helpful to establish the criteria for more practical screening of the complex extracts of sage species.

## 4. Conclusions

Our study provides data on the chemical profile and potential bioactivities of ten Moldavian *Salvia* species from ex situ crop cultures (*S. officinalis*, *S. sclarea*, *S. tesquicola*, *S. aethiopis*, *S. austriaca*, *S. kopetdaghensis*, *S. nemorosa*, *S. nutans*, *S. pratensis*, *S. verticillata*). To the best of our knowledge, this is the first study on Moldavian sage plants from field crops regarding non-volatile chemical composition and biological activity. Moreover, data about LC-HRMS/MS-based phytochemical profiling of *S. kopetdaghensis* and *S. nutans* are reported herein for the first time. *S. officinalis*, *S. sclarea*, *S. kopetdaghensis,* and *S. verticillata* are valuable sources of polyphenols. LC-HRMS/MS metabolite profiling indicated a rich composition of sage plants, including eight chemical classes (phenolic acids, flavonoids, diterpenes, sesterpenes, triterpenes, organic acids, fatty acids, and sugars). *S. aethiopis* presented the most complex profile. Hydroxybenzoic acids, hydroxycinnamic acids, and their oligomers were identified in sage species having a specific distribution. Caffeic and rosmarinic acids were ubiquitously distributed in all *Salvia* species. Salvianolic acids were primarily found in *S. officinalis*, *S. tesquicola*, *S. nemorosa*, *S. kopetdaghensis*, and *S. pratensis*. Luteolin, apigenin, and quercetin derivatives were the main flavonoids identified, but the composition varied between the tested sages. Luteolin derivatives were present in all species, with luteolin-7-*O*-glucuronide being the most common flavonoid. Rosmanol and carnosol derivatives were the main diterpenes in the investigated Moldavian sage plants. Among the triterpenes, oleanolic and ursolic acids were present in *S. nemorosa*, *S. nutans*, *S. verticillata*, and *S. tesquicola*. Multivariate analysis showed that Moldavian *S. officinalis*, *S. austriaca,* and *S. nutans* formed individual clusters. All analyzed sages scavenged free radicals and acted as reducing agents, with *S. officinalis*, *S. verticillata,* and *S. kopetdaghensis* being the most effective antioxidants. The highest antimicrobial activity was found for *S. officinalis*. The tested *Salvia* species (*S. officinalis*, *S. sclarea*, *S. tesquicola*, *S. aethiopis*, *S. kopetdaghensis*) did not show cytotoxic properties on breast cancer cell lines (MCF-7 and MDA-MB-231) On the contrary, the extracts seemed to show proliferative activity on tested cell lines. In this regard, for a substantiated statement, it is necessary to investigate a broader range of doses and variations of the exposure period.

Our research contributes to knowledge about the chemistry and biological potential of understudied Moldavian *Salvia* species, providing evidence for future studies that can lead to developing sage-based health-promoting agents in oxidative stress-related disturbances and redox medicine.

## Figures and Tables

**Table 1:** Identification, extraction data, and total phenolic content of *Salvia* species.

No.	Species	Voucher	Code	Extraction Yield (%)	TPC (mg GAE/g Extract)
1	*Salvia sclarea* L.	SS/2019	SS	53.35	110.90 ± 0.26
2	*Salvia tesquicola* Klok. and Pobed.	ST/2019	ST	51.39	71.47 ± 0.16
3	*Salvia aethiopis* L.	SE/2019	SE	47.71	81.43 ± 0.25
4	*Salvia nutans* L.	SNu/2019	SNu	55.11	66.12 ± 0.15
5	*Salvia verticillata* L.	SV/2019	SV	48.52	107.62 ± 0.08
6	*Salvia officinalis* L.	SO/2019	SO	55.33	126.91 ± 0.56
7	*Salvia nemorosa* L.	SNe/2019	SNe	47.56	98.42 ± 0.38
8	*Salvia pratensis* L.	SP/2019	SP	52.35	81.70 ± 0.20
9	*Salvia austriaca* Jacq.	SA/2019	SA	47.67	57.87 ± 0.33
10	*Salvia kopetdaghensis* Kudr.	SK/2019	SK	48.67	107.63 ± 0.21

**Table 2:** LC-HRMS/MS identification of specialized metabolites in *Salvia* species.

No.	Proposed Identity	Class	T_R_ (min)	Exp. (*m*/*z*)	Calcd. (*m*/*z*)	Δ (ppm)	MF	MS/MS (-)	Ref.	Samples ^§^
**1**	Sucrose	Sugars	1.8	341.1104	341.1089	−4.28	C_12_H_22_O_11_	179.0548, 119.0359	[28]	SS, ST, SE, SNu, SV, SO, **SNe**, SP, SA, **SK**
**2**	Malic acid	Organic acid	2.4	133.0145	133.0142	−1.89	C_4_H_6_O_5_	115.0093	[41]	**SE**, **SNu**, SV, SO, SP, SA
**3**	Quinic acid	Organic acid	4.7	191.0567	191.0561	−3.06	C_7_H_12_O_6_	145.0453, 129.0482, 115.0363, 101.0573	[41]	SNu, SK
**4**	Danshensu/salvianic acid	Phenolic acid	6.3	197.0456	197.0455	−0.27	C_9_H_10_O_5_	179.0395, 135.0461, 123.0456	[25]	SS, ST, SE, SV, SO, SNe, SP, SK
**5**	Dihydroxybenzoic acid	Phenolic acid	7.9	153.0124	153.0193	6.05	C_7_H_6_O_4_	108.0225	[41]	SNe
**6**	Hydroxybenzoic acid	Phenolic acid	10.3	137.0249	137.0244	−3.49	C_7_H_6_O_3_	108.0185	[30]	SS, ST, SE, SV, SO, SNe, SP, SK
**7**	Tuberonic acid-*O*-hexoside	Fatty acid	15.2	387.1679	387.1661	−4.75	C_18_H_8_O_9_	207.0956, 163.1025, 101.0232	[40]	ST, SNu, SNe
**8**	Caffeic acid *	Phenolic acid	17.3	179.0359	179.0350	−5.10	C_9_H_8_O_4_	161.0434, 135.0470, 107.0488	[25]	SS, ST, **SE**, **SNu**, SV, SO, SNe, SP, SA, SK
**9**	Caffeoylthreonic acid	Phenolic acid	18.5	297.0645	297.0616	3.66	C_13_H_14_O_4_	179.0326, 161.0227, 135.0323	[36]	SNe, SP
**10**	Apigenin-*O*-pentoside-*O*-hexoside	Flavonoid	21.1	563.1428	563.1406	−3.85	C_26_H_28_O_14_	473.1105, 383.0745, 353.0694, 297.0852, 269.0640	[40]	SNu
**11**	Luteolin-*O*-hexoside-*O*-glucuronide	Flavonoid	21.7	623.1243	623.1254	1.72	C_27_H_28_O_17_	447.0639, 285.0218	[28]	ST
**12**	Luteolin di-*O*-glucuronide	Flavonoid	22.0	637.1065	637.1046	−2.92	C_27_H_26_O_18_	351.0358, 285.0122	[28]	ST, SE, **SNe**, SA
**13**	Quercetin-*O*-hexoside	Flavonoid	22.1	463.0902	463.0902	−4.31	C_21_H_20_O_12_	301.0424, 300.0286	[29]	SO
**14**	Caffeic acid-*O*-hexoside	Phenolic acid	22.4	341.0914	341.0878	1.18	C_15_H_18_O_9_	223.0618, 179.0737, 135.0311	[39]	SNu, SO
**15**	Luteolin-*O*-hexoside-*O*-rhamnoside	Flavonoid	22.9	593.1525	593.1512	−2.20	C_27_H_30_O_15_	327.0822, 285.0445, 267.0343	[28]	ST, SNu, SO
**16**	Quercetin-*O*-rhamnoside-*O*-glucoside	Flavonoid	23.2	609.1473	609.1461	−1.96	C_27_H_30_O_16_	300.0167, 271.0154, 150.994	[28]	SNu, SNe, SK
**17**	Luteolin−7-*O*-glucoside *	Flavonoid	23.7	447.0945	447.0933	−2.71	C_21_H_20_O_11_	285.0434, 257.0504, 151.0031	[28]	SS, ST, **SE**, SNu, SV, SO, **SNe**, SP, **SA**, SK
**18**	Feruloylmalic acid	Phenolic acid	24.2	309.0612	309.0616	1.26	C_14_H_13_O_8_	193.0522, 133.0381	[38]	SE
**19**	12-Deoxy-7,7-dimethoxy-6-ketoroyleanone	Diterpene	24.3	373.2030	373.2020	−2.55	C_22_H_30_O_5_	358.1920, 343.1934, 283.1752	[38]	ST, **SNu**, SNe
**20**	Luteolin-*O*-glucuronide I	Flavonoid	24.9	461.0732	461.0725	−1.41	C_21_H_18_O_12_	357.0635, 285.0393, 175.0150	[31]	SS, ST, SE, SV, SO, SNe, SP, SA, **SK**
**21**	Apigenin-7-*O*-glucoside *	Flavonoid	25.5	431.1002	431.0984	−4.23	C_21_H_20_O_10_	269.0631, 151.0096	[28]	SS, SNe, SA
**22**	Gallocatechin	Flavonoid	25.6	305.0652	305.0667	4.82	C_15_H_14_O_7_	225.1161	[42]	**SNu**, SO, SNe, SA
**23**	Rosmarinic acid *	Phenolic acid	26.5	359.0786	359.0772	−3.77	C_18_H_16_O_8_	197.0494, 179.0375, 161.0261, 135.0451	[28]	**SS**, **ST**, **SE**, SNu, **SV**, **SO**, **SNe**, **SP**, SA, **SK**
**24**	Salvianolic acid B	Phenolic acid	27.4	717.1492	717.1461	−4.30	C_36_H_30_O_16_	519.0998, 493.1205, 295.0849, 203.0513, 179.0488	[28]	SS, ST, SO, SNe, SP, **SK**
**25**	Luteolin-*O*-glucuronide II	Flavonoid	28.2	461.0742	461.0725	−3.57	C_21_H_18_O_12_	285.0589, 241.0651, 199.0513, 151.0129, 133.0326	[31]	SS
**26**	Salvianolic acid K	Phenolic acid	28.4	555.1153	555.1144	−1.59	C_27_H_24_O_13_	537.1068, 493.1148, 359.0808, 197.0465	[28]	ST, SV, SO, SNe, SA, SK
**27**	Salvianolic acid H	Phenolic acid	28.6	537.1073	537.1038	−6.41	C_27_H_22_O_12_	493.1205, 359.0827, 295.0642, 161.0271	[25]	SP
**28**	Luteolin-*O*-acetylglucuronide	Flavonoid	28.5	503.0901	503.0831	−0.57	C_23_H_20_O_13_	285.0435, 217.0504, 175.0343	[31]	SNu, SV
**29**	Methylrosmarinate	Phenolic acid	29.4	373.0909	373.0929	5.32	C_31_H_52_O_11_	193.0584, 179.0347, 161.0173, 135.0479	[28]	ST, SE, SV, SO, **SNe**, **SP**, SK
**30**	Chrysoeriol-*O*-acetylglucuronide I	Flavonoid	30.6	517.1053	517.0988	−2.96	C_24_H_22_O_13_	299.0594, 217.0362, 175.0267	[31]	SNu
**31**	Luteolin *	Flavonoid	30.8	285.0407	285.0405	−0.83	C_15_H_10_O_6_	151.0060, 133.0314, 107.0130	[28]	**SS**, ST, SO, SP, **SA**, SK
**32**	Trihydroxyoctadecadienoic acid	Fatty acid	31.8	327.2187	327.2177	−3.05	C_18_H_32_O_5_	309.2011, 239.1382, 229.1456, 211.1312, 171.1039	[28]	SS, **ST**, **SE**, SNu, **SV**, SO, SNe, SP, SA, SK
**33**	Chrysoeriol-*O*-acetylglucuronide II	Flavonoid	32.6	517.1053	517.0988	−2.96	C_24_H_22_O_13_	457.0739, 299.0594, 284.0272, 217.0362, 175.0267	[31]	SNu
**34**	Tricoumaroylspermidine	Phenolic acid	33.1	582.2623	582.2610	−2.3	C_34_H_37_N_3_O_6_	462.2030, 342.1455, 316.1757, 145.0278, 119.0521	[24]	SNe, SP, SA, SK
**35**	Trihydroxyoctadecenoic acid	Fatty acid	33.5	329.2335	329.2333	−0.76	C_18_H_34_O_5_	229.1436, 211.1309, 171.1004	[40]	ST, SE, SNu, **SV**, SO, SNe, SP, SK
**36**	Apigenin	Flavonoid	33.7	329.0678	329.0667	−3.40	C_17_H_14_O_7_	314.0447, 299.0225, 285.0452, 271.0294, 243.0318, 227.0390	[31]	**SS**, **SA**
**37**	Hydroxycarnosic acid I	Diterpene	34.5	347.1858	347.1864	1.72	C_20_H_28_O_5_	303.2015, 259.2180	[40]	**SS**, ST
**38**	Dihydroxyhexadecanoic acid	Fatty acid	34.9	287.2240	287.2228	−4.22	C_16_H_32_O_4_	171.1045	[40]	SV, SO, SP, SA, SK
**39**	Hydroxyoxooctadecadienoic acid	Fatty acid	36.0	309.2080	309.2071	−2.79	C_18_H_30_O_4_	291.1957, 251.1660, 171.1045	[40]	SS, SNu, SNe, SP, SA
**40**	Rosmanol I	Diterpene	36.4	345.1709	345.1707	−0.44	C_20_H_26_O_5_	330.1366, 315.1609, 301.1794, 283.1413	[34]	ST, SO, SNe
**41**	Cirsimaritin	Flavonoid	37.3	313.0716	313.0718	0.51	C_17_H_14_O_6_	289.0486, 283.0281, 255.0338, 227.0375, 163.0053, 135.0085, 117.0363	[27]	**SS**, ST, SE, SO, SNe, SP, **SA**
**42**	Rosmanol II	Diterpene	37.6	345.1717	345.1707	−2.75	C_20_H_26_O_5_	330.1366, 315.1609, 301.1794, 283.1413	[34]	SO
**43**	Lachnocalyxolide C	Sesterpene	38.0	461.2562	461.2545	−3.73	C_26_H_38_O_7_	429.2174, 385.2302, 341.2410	[43]	SNu
**44**	Salvimirzacolide I	Sesterpene	38.5	417.2630	417.2646	3.94	C_25_H_38_O_5_	373.2700, 235.1544, 205.1478, 137.0943	[38]	SE
**45**	Rosmanol III	Diterpene	38.7	345.1698	345.1707	2.74	C_20_H_26_O_5_	330.1366, 315.1609, 301.1794, 283.1413	[34]	**SO**, SP
**46**	Lachnocalyxolide C’	Sesterpene	39.3	461.2581	461.2545	−7.84	C_26_H_38_O_7_	385.2133, 341.2381	[43]	SNu
**47**	Hydroxycarnosic acid II	Diterpene	40.0	347.1858	347.1864	1.72	C_20_H_28_O_5_	329.1832, 303.2018, 259.2078	[40]	**ST**, SNe, SK
**48**	Genkwanin	Flavonoid	40.6	283.0620	283.0612	−2.83	C_16_H_12_O_5_	268.0311, 240.03666, 239.0341, 211.0332,	[34]	SS, SP, SA
**49**	Hydroperoxyoctadecadienoic acid	Fatty acid	40.7	311.2237	311.2228	−2.94	C_18_H_32_O_4_	293.2085, 253.1793, 223.1693	[40]	ST, SNu, SV, SO, SP, SA
**50**	Lachnocalyxolide A	Sesterpene	42.2	429.2305	429.2283	−5.20	C_25_H_34_O_6_	385.2405, 341.2499, 299.2342, 205.1180	[43]	SNu
**51**	Acetylhorminone II	Diterpene	42.7	373.1995	373.2020	5.74	C_22_H_30_O_5_	313.1384, 193.1266	[38]	SV
**52**	Carnosic acid	Diterpene	42.8	331.1935	331.1915	−4.87	C_20_H_28_O_4_	287.2177, 259.2130	[34]	**ST**, SO, SNe, SK
**53**	Hydroxydodecanoic acid	Fatty acid	42.9	215.1639	215.1653	6.33	C_12_H_24_O_3_	171.1045	[40]	SA
**54**	Methoxycarnosol	Diterpene	44.5	359.1856	359.1864	2.21	C_21_H_28_O_5_	283.1678	[31]	SO
**55**	Dihydroxycarnosic acid	Diterpene	44.9	363.1834	363.1813	−5.73	C_20_H_28_O_6_	319.1970, 275.2060, 257.1916	[31]	ST, **SK**
**56**	Rosmadial	Diterpene	45.3	343.1543	343.1551	2.32	C_20_H_24_O_5_	299.1693, 243.1035, 216.0784	[34]	**SO**, SNe, SP, SA
**57**	Salvimirzacolide I	Sesterpene	45.7	417.2654	417.2646	−1.80	C_25_H_38_O_5_	373.2784, 235.1751, 137.1009	[38]	SE
**58**	Salvipiliferol	Diterpene	46.1	303.1973	303.1966	−2.41	C_19_H_28_O_3_	205.1221	[38]	SNe
**59**	Carnosol	Diterpene	46.5	329.1761	329.1758	−0.81	C_20_H_26_O_4_	285.1871, 201.0936	[34]	**SO**, SK
**60**	Hydroxyoctadecatrienoic acid	Fatty acid	46.9	293.2125	293.2122	−0.96	C_18_H_30_O_3_	275.2143, 224.1450, 195.1416	[40]	**SS**, ST, SE, **SNu**, **SV**, **SNe**, **SP**, SA, SK
**61**	*ent*-19-Acetoxy-15,16-epoxy-3,13(16),14-clerodatrien-6,18-diol	Diterpene	48.2	375.2177	375.2177	5.31	C_22_H_32_O_5_	315.2082, 285.2014	[38]	**SV**, SK
**62**	Hydroxysalviol	Diterpene	48.3	317.2114	317.2122	2.57	C_20_H_30_O_3_	273.2682, 137.1239	[40]	SNe
**63**	Oxooctadecatrienoic acid	Fatty acid	48.4	291.1980	291.1966	−2.16	C_18_H_28_O_3_	211.1170, 109.1020	[40]	SA
**64**	Acetylhorminone III	Diterpene	48.7	373.2028	373.2020	−2.01	C_22_H_30_O_5_	313.1384, 193.1266	[38]	SO
**65**	Rosmaridiphenol	Diterpene	49.0	315.1958	315.1966	2.43	C_20_H_28_O_3_	285.1877, 201.0888	[31]	SO
**66**	Hydroxytetradecanoic acid	Fatty acid	49.6	243.1974	243.1966	−3.41	C_14_H_28_O_3_	197.1966	[40]	SA
**67**	Hydroxyoctadecadienoic acid	Fatty acid	49.7	295.2269	295.2279	3.27	C_18_H_32_O_3_	277.2134, 235.1680, 195.1328, 171.1023	[40]	SS, ST, SNu, SV, SO, SNe, **SP**, SK
**68**	Methylcarnosic acid	Diterpene	51.6	345.2084	345.2071	−3.66	C_21_H_30_O_4_	301.2190, 286.2012, 191.1768	[34]	ST, SV, **SO**, SNe, SP
**69**	Hydroxyhexadecanoic acid I	Fatty acid	52.7	271.2260	271.2279	−0.12	C_16_H_32_O_3_	225.2151	[40]	SP, **SA**, SK
**70**	Salviol	Diterpene	52.9	301.2095	301.2173	−1.31	C_20_H_30_O_2_	205.1268, 169.9510	[24]	SNe
**71**	Hydroxyhexadecanoic acid II	Fatty acid	53.4	271.2270	271.2279	3.19	C_16_H_32_O_3_	225.2151	[40]	SP, SK
**72**	Oleanolic acid	Triterpene	54.1	455.3549	455.3531	1.25	C_30_H_48_O_3_	407.3436	[34]	ST, SNu, SV, SNe, SP, SA
**73**	Ursolic acid	Triterpene	54.6	455.3551	455.3531	−4.45	C_30_H_48_O_3_	408.3315, 373.2988	[34]	ST, SNu, SV, SO, SNe, SP

* Identified by standard injection; ^§^ sample code as in Table 1; T_R_, retention time; Δ, mass error; MF, molecular formula; MS, mass spectra; in bold the most abundant compounds.

**Table 3:** Antimicrobial activity of *Salvia* species.

	Sample ^§^	SS	ST	SE	SNu	SV	SO	SNe	SP	SA	SK
Microbial Strain		MIC (mg/mL)									
*S. aureus* ATCC 25923		1.25	2.5	1.25	5	1.25	0.625	2.5	2.5	2.5	2.5
*S. pneumoniae* ATCC 49619		1.25	2.5	2.5	2.5	2.5	0.625	2.5	2.5	2.5	2.5
*E. coli* ATCC 25922		5	5	5	5	2.5	5	5	5	5	2.5
*P. aeruginosa* ATCC 27853		2.5	2.5	2.5	2.5	2.5	2.5	2.5	2.5	2.5	2.5
*C. albicans* ATCC 10231		2.5	2.5	5	5	2.5	2.5	1.25	1.25	5	2.5

^§^ Sample codes as in Table 1; MIC, minimum inhibitory concentration.

**Table 4:** Antioxidant activity of *Salvia* species.

	Sample ^§^	DPPH	ABTS	FRAP
Test		EC_50_ (μg/mL)		
**SS**		32.23 ± 0.35 ^a^	17.20 ± 0.10 ^a^	29.67 ± 0.02 ^a^
**ST**		41.16 ± 0.15 ^b^	26.50 ± 0.20 ^b^	28.51 ± 0.22 ^b^
**SE**		42.00 ± 0.10 ^b^	17.00 ± 0.10 ^a^	36.94 ± 0.18 ^c^
**SNu**		178.90 ± 1.1 ^c^	50.83 ± 0.15 ^c^	52.08 ± 0.01 ^d^
**SV**		27.36 ± 0.32 ^d^	13.40 ± 0.10 ^d^	19.75 ± 0.02 ^e^
**SO**		25.33 ± 0.05 ^d^	8.13 ± 0.05 ^e^	21.01 ± 0.02 ^e^
**SNe**		57.40 ± 0.40 ^e^	16.46 ± 0.15 ^f^	55.61 ± 0.33 ^f^
**SP**		39.53 ± 0.15 ^f^	15.06 ± 0.05 ^g^	40.94 ± 0.07 ^g^
**SA**		146.6 ± 1.1 ^c^	59.16 ± 0.05 ^h^	80.02 ± 0.05 ^h^
**SK**		38.53 ± 0.25 ^f^	14.06 ± 0.05 ^i^	19.74 ± 0.09 ^e^
**Gallic acid**		1.60 ± 0.01 ^g^	0.60 ± 0.01 ^j^	1.57 ± 0.01 ^i^

^§^ Sample codes as in Table 1; results are presented as mean ± SD from three replicates; different superscript letters indicate significant differences at *p* < 0.05.

## Data Availability

Data is contained within the article.
